# Automatic image generation and stage prediction of breast cancer immunobiological through a proposed IHC-GAN model

**DOI:** 10.1186/s12880-024-01522-y

**Published:** 2025-01-06

**Authors:** Afaf Saad, Noha Ghatwary, Safa M. Gasser, Mohamed S. ElMahallawy

**Affiliations:** 1 Electronics and Communications, Arab Academy for Science, Heliopolis, Cairo, 2033 Egypt; 2 Department of Computer Engineering, Arab Academy for Science, Smart Village, Giza, 2033 Egypt; 3 Department of Electrical and Communications, The British University in Egypt, El Sherouk, Cairo, 11837 Egypt

**Keywords:** Breast cancer, IHC, GAN, Pix2Pix

## Abstract

Invasive breast cancer diagnosis and treatment planning require an accurate assessment of human epidermal growth factor receptor 2 (HER2) expression levels. While immunohistochemical techniques (IHC) are the gold standard for HER2 evaluation, their implementation can be resource-intensive and costly. To reduce these obstacles and expedite the procedure, we present an efficient deep-learning model that generates high-quality IHC-stained images directly from Hematoxylin and Eosin (H&E) stained images. We propose a new IHC-GAN that enhances the Pix2PixHD model into a dual generator module, improving its performance and simplifying its structure. Furthermore, to strengthen feature extraction for HE-stained image classification, we integrate MobileNetV3 as the backbone network. The extracted features are then merged with those generated by the generator to improve overall performance. Moreover, the decoder’s performance is enhanced by providing the related features from the classified labels by incorporating the adaptive instance normalization technique. The proposed IHC-GAN was trained and validated on a comprehensive dataset comprising 4,870 registered image pairs, encompassing a spectrum of HER2 expression levels. Our findings demonstrate promising results in translating H&E images to IHC-equivalent representations, offering a potential solution to reduce the costs associated with traditional HER2 assessment methods. We extensively validate our model and the current dataset. We compare it with state-of-the-art techniques, achieving high performance using different evaluation metrics, showing 0.0927 FID, 22.87 PSNR, and 0.3735 SSIM. The proposed approach exhibits significant enhancements over current GAN models, including an 88% reduction in Frechet Inception Distance (FID), a 4% enhancement in Learned Perceptual Image Patch Similarity (LPIPS), a 10% increase in Peak Signal-to-Noise Ratio (PSNR), and a 45% reduction in Mean Squared Error (MSE). This advancement holds significant potential for enhancing efficiency, reducing manpower requirements, and facilitating timely treatment decisions in breast cancer care.

## Introduction

Breast cancer remains one of the most prevalent cancers affecting women globally [1]. According to the latest statistics, In 2023, it is estimated that the United States will experience about 1,958,310 new cancer cases and 609,820 cancer-related mortalities, with breast cancer significantly contributing to these statistics [2]. Breast cancer incidence has steadily increased, with rates rising by 0.5% per year from 2010 to 2019, mainly due to localized-stage and hormone receptor-positive cases [3]. The implementation of early detection strategies has significantly impacted breast cancer mortality rates. Since reaching their peak in 1989, breast cancer mortality rates have consistently decreased, falling by 43% by 2020. This reduction corresponds to around 460,000 fewer breast cancer deaths over the years [3]. Early detection, primarily through regular mammography screening, has enabled the identification of breast cancer at earlier, more treatable stages, thereby improving survival rates and reducing mortality.

**Fig. 1:**
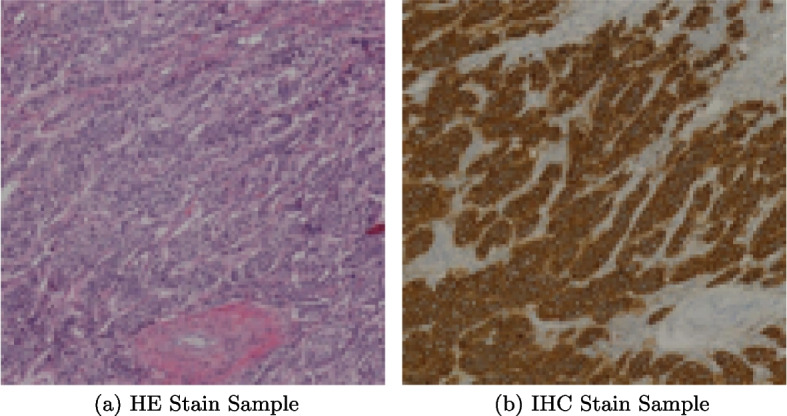
Sample from the HE and IHC stain image

Determining the stage of breast cancer is pivotal for developing an appropriate treatment plan. One of the key biomarkers in this process is the Human Epidermal Growth Factor Receptor 2 (HER2). HER2 status is typically assessed through a biopsy, where tissue samples are stained using hematoxylin and eosin (HE) stain, followed by immunohistochemical (IHC) staining to determine HER2 expression levels. The two different stains are illustrated in Fig. 1. IHC staining categorizes HER2 expression into four levels, critical for guiding treatment decisions. However, this process is costly and labor-intensive, requiring significant observation and expertise to interpret the results accurately [4, 5].

In recent years, medical imaging has witnessed a remarkable transformation with the advent of deep learning [6–10]. By employing advanced algorithms, deep learning can efficiently and effectively produce reliable HER2 expression images, facilitating quicker and more accurate diagnoses [11]. Breast cancer diagnostics emphasizes generating IHC images from H&E stained images using deep learning to streamline and enhance the diagnostic process, reducing time and costs significantly. This technological advancement improves diagnostic accuracy and substantially reduces costs and labor associated with traditional methods [12]. There are various image translation methods in the literature that utilize deep learning to generate IHC images. An innovative pyramid Pix2Pix model was proposed by Liu et al. [13] to generate IHC images for breast cancer. This model successfully converted H&E images into IHC images while providing a reliable tool for HER2 expression evaluation. Moreover, Roy et al. [14] developed a model named CGNReg, that synthesizes IHC images from H&E slides and aligns them with real IHC images, addressing large image scales and histological changes. Zhu et al. [15] discussed five methods that have a high superior performance in image translation of IHC images from H&E images using the Breast Cancer Immunohistochemical (BCI) dataset. Additionally, Liu et al. [16] proposed MGGAN, which enhances breast cancer image detail translation by integrating both low and high-frequency components of H&E images, outperforming other synthesis methods. In [17], new approach was proposed in the form of TC-CycleGAN, which incorporated texture constraints and a self-attention mechanism, significantly improved the quality of virtual stained sections, and showcased the potential of virtual staining technology in clinical applications. Furthermore, using an Adaptive Supervised PatchNCE [18] loss for translating H&E stain to IHC improved performance over existing methods by dealing with input-to-target inconsistencies directly. Despite advancements, some methods fail to consider color features in pathology images or accurately reflect image quality through the Peak-Signal-to-Noise Ratio (PSNR) and Structural similarity index measure (SSIM), limiting their clinical application.

In this study, to overcome the aforementioned challenges, we propose an IHC-GAN model that enhances the Pix2PixHD [19] through several modifications to effectively translate H&E staining to IHC staining. The main contributions of our work are summarized as follows:

- We employ a fine-tuned MobileNetV3 Classifier to categorize input images into 0, 1+, 2+, or 3+ levels to provide informative labels that guide the image generation process.
- The suggested IHC-GAN is a two-scale generator model that uses a trained classifier’s dual-scale feature sharing to get fine and coarse details, making the feature representation more complete. Additionally, it incorporates the Adaptive Instance Normalization (AdaIN) in the decoders to adjust the style of generated synthesized images based on classifier labels, ensuring accurate reflection of the desired staining style and consistent color and texture.
- We utilize Inverted Residual Blocks to significantly and computationally reduce the number of parameters to develop a faster model. This not only facilitates better gradient flow during training but also allows us to capture more complex patterns with fewer resources, thereby enhancing the efficiency of our image-generation process when compared to baseline models.
- We extensively validate the proposed model on the Breast Cancer Immunohistochemical (BCI) dataset, and We use several assessments to provide a proper assessment of the quality of the generated or synthesized images from the perceptual and quantitative points of view.

This paper is organized as follows: Proposed methodology section describes implementing our proposed IHC-GAN model for image generation. Loss function section focuses on the loss function used in the model. In Dataset section, the dataset and preprocessing steps are described. This is followed by Performance evaluation section, which presents the performance evaluation using various metrics. Finally, Results and discussion section provides the experimental results and discussion.

## Proposed methodology

**Fig. 2:**
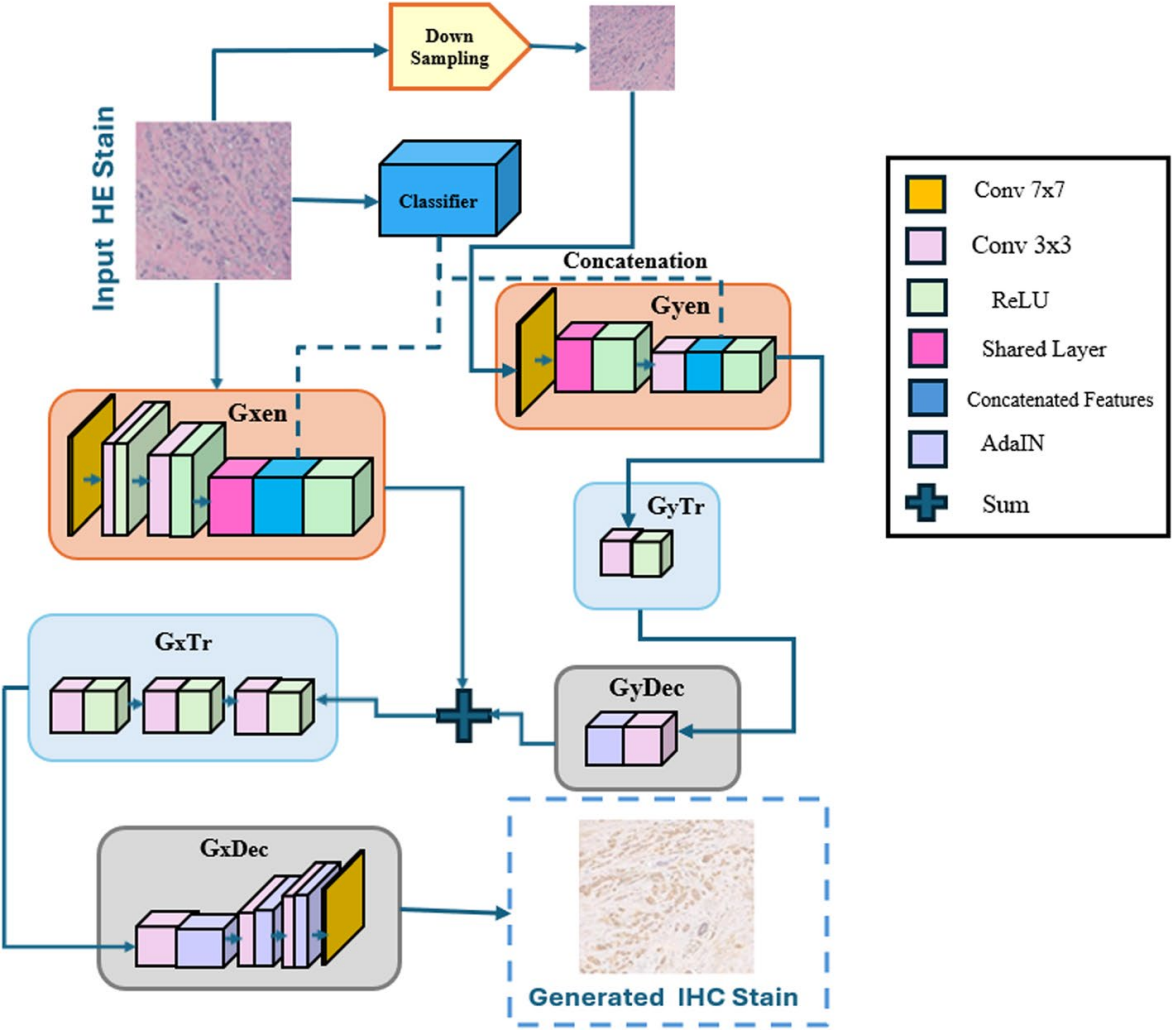
Overview of the IHC-GAN Model Architecture. The input HE stain image undergoes down-sampling and feature extraction through a classifier. The extracted features are then concatenated and passed through various components, including the encoder modules (GxEn and GyEn), transformation layers (GxTr and GyTr), and decoder modules (GxDec and GyDec). These modules utilize a combination of 7x7 and 3x3 convolutional layers, ReLU activations, and shared feature layers. The Adaptive Instance Normalization (AdaIN) is used to match the style of the generated IHC image to the desired output

In this section, we introduce our proposed IHC-GAN model to translate the H&E images into IHC images as illustrated in Fig. 2. The workflow of the proposed model consists of several phases, *Pre-processing, Classification, Generator* and *Discriminator*. In our model, the first step is *Pre-processing* to prepare the input data for analysis and interpretation. Next, *Classification* involves identifying the characteristics of an object that fit into a specific category based on the training set used in image analysis. The first generator (G1) employs the MobileNetV3 classifier for the classification process and uses its extracted features. At the same time, the image is downscaled and passed through a second generator (G2). The features from G2 are then integrated into G1, allowing the generation of the final synthesized image.

### Pre-processing phase

As a preprocessing phase, we normalize the data so that the pixel values fall into the [−1, 1] range to satisfy neural network requirements. This significantly facilitates and expedites the training process. We employ various data augmentation techniques, such as random rotations and flips, to artificially expand the training dataset and introduce variability. Moreover, due to limitations of High-Performance Computing (HPC) devices, we down-sample input images to 256$\times$256. We initially resize The images from 1024$\times$1024 to 256$\times$256 to reduce the data size, thereby challenging the generation of high-quality images from low-resolution ones.

### Classification phase

The generator employs the *MobileNetV3* [20] classifier to determine the IHC levels from the H&E stain input images in our model. This allows it to create a specific stain for each generated IHC image. MobileNetV3 classifies the input H&E images into four classes: 0, 1+, 2+, and 3+ levels. Then we use these labels in the generator phase to direct the images’ coloring to the suitable stain, ensuring that each image is accurately and efficiently processed according to its classification.

**Fig. 3:**
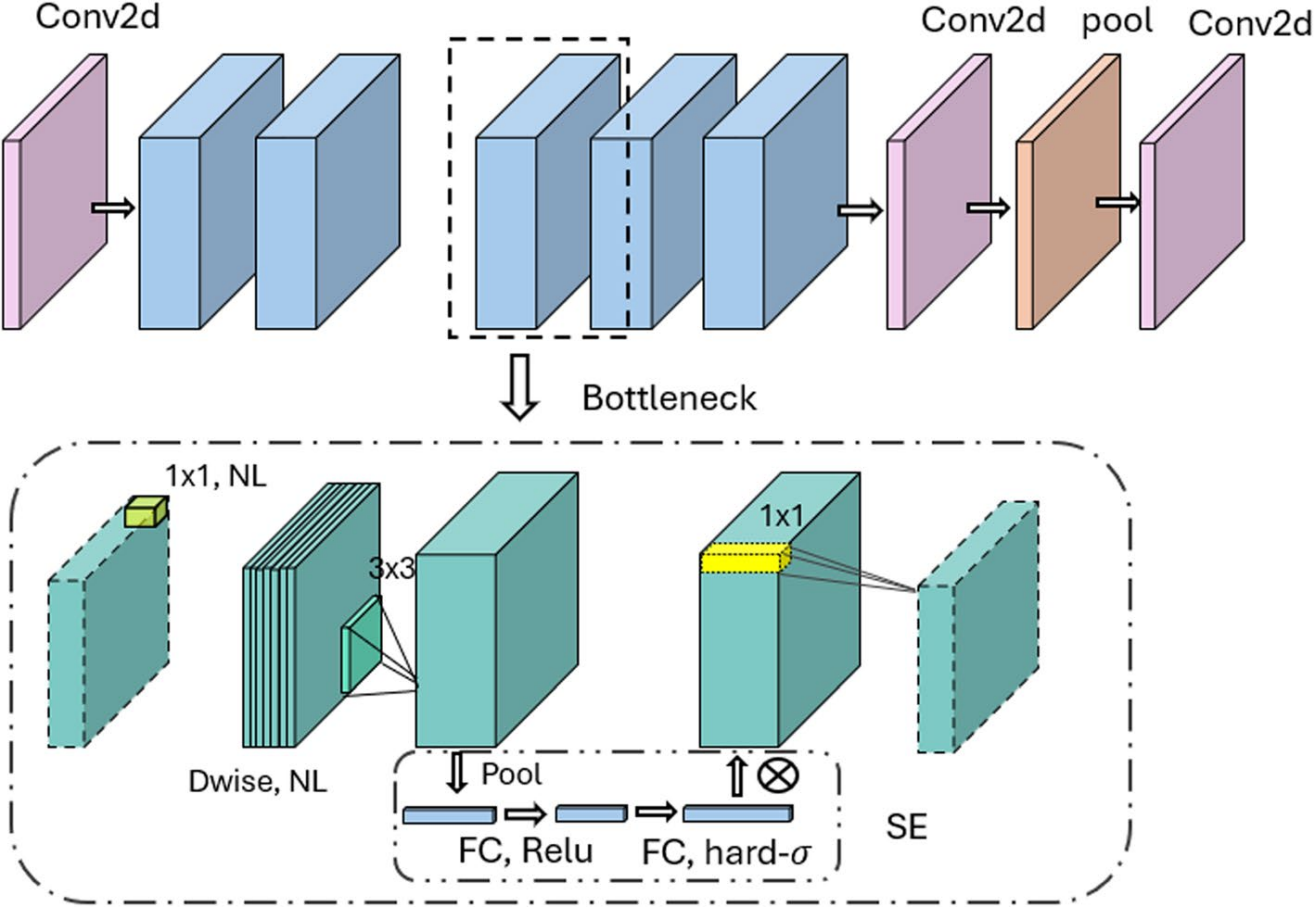
Architecture of MobileNetv3 Network. The architecture begins with a 2D convolutional layer (Conv2d), followed by a bottleneck structure that compresses the input features using depthwise separable convolutions (Dwise). Non-linearity (NL) activations are applied after each convolutional layer. The bottleneck block includes a 3x3 convolution and pooling operations. In the Squeeze-and-Excitation (SE) block, global pooling is applied, and the features are passed through fully connected (FC) layers with ReLU and hard-sigmoid activations. The SE block adaptively reweights the feature channels, enhancing model performance and efficiency

The MobileNetV3 demonstrates superior performance compared to other classifiers (i.e. ResNet18, ResNet50, ResNet100, VGG16, VGG19, MobileNetV1, and MobileNetV2, MobileNetV3), achieving an impressive accuracy of 96%. MobileNetV3’s architecture, as presented in Fig. 3, is optimized for mobile and embedded applications, balancing latency, size, and accuracy. Key features include complex SWISH activation functions and squeeze-and-excitation modules in the MBConv blocks, contributing to its efficiency and performance. Compared to other classifiers, it is more efficient due to its reduced number of parameters and computations without compromising accuracy. These design choices make MobileNetV3 accurate and fast [21].

### Generator phase

As shown in the proposed model in Fig. 2. We propose Dual-scale generators Gx and Gy, consisting of three parts: an encoder, a transformer, a bottleneck, and a decoder. Using dual-scale generators is effective because they capture fine-grained details and high-level structures in the input data, enabling the model to generate visually appealing and structurally coherent images, as mentioned in [19].

#### Coupled encoders

The first encoder processes input images of size 256$\times$256$\times$3 and down-samples them to patches of size 64$\times$64$\times$3. This encoder starts with a 7$\times$7 convolutional layer, followed by three downsampling layers with 3$\times$3 kernels and a stride of 2, resulting in feature maps of size 32$\times$32. Similarly, the second encoder, on the other hand, processes input image patches of size 64$\times$64$\times$3 also starts with a 7$\times$7 convolutional layer but undergoes two downsampling operations, producing feature maps of size 16$\times$16. Each convolution layer is followed by instance normalization and a leaky ReLU activation function to ensure stable training and efficient gradient flow.

These two encoders share some of their layers, which reduces the overall number of parameters and is particularly useful in scenarios with limited computing resources or when training large-scale models. This layer-sharing mechanism optimizes resource usage and maintains continuity and unity across different scales, contributing to the generation of coherent images [22]. After reshaping, we concatenate the feature maps from the two encoders with those from a classifier network, specifically MobileNetV3. This approach leverages the rich, pre-learned features from MobileNetV3, enhancing the overall feature representation and improving the model’s generalization capabilities.

#### Transformer

**Fig. 4:**
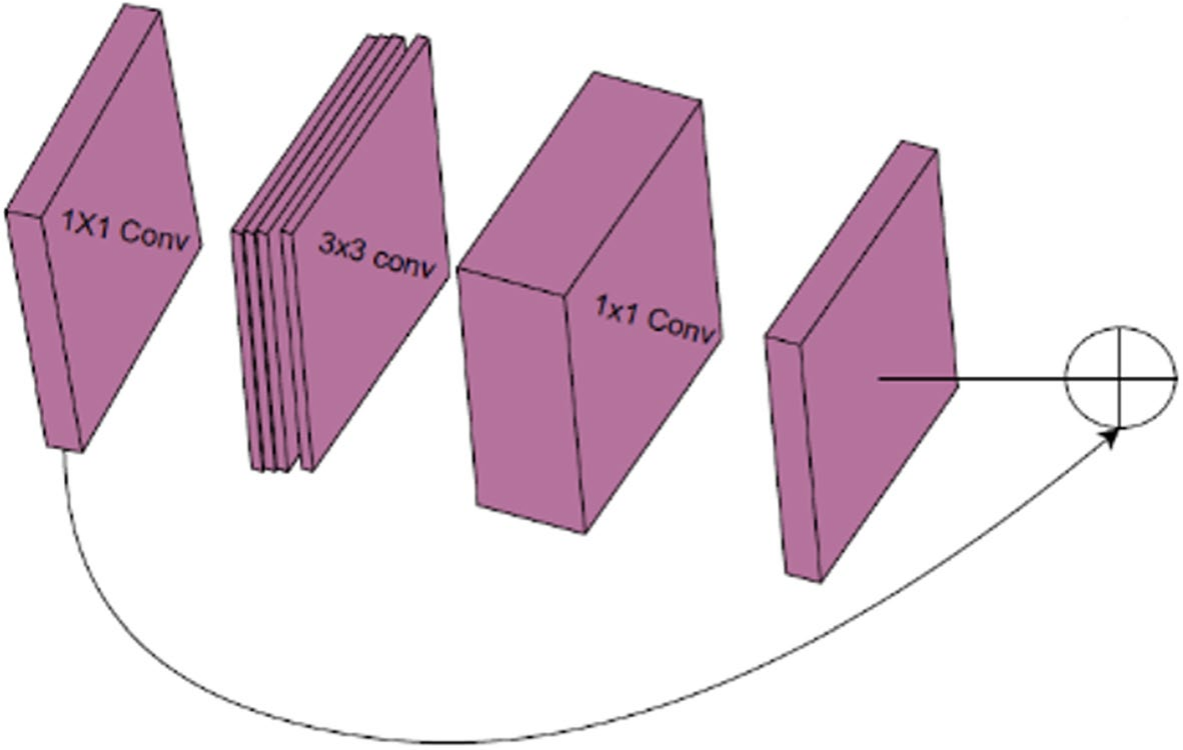
Inverted Residual Block

After concatenation, we process the results using a transformer consisting of an inverted residual block. The key idea is to replace a full convolutional operator with its factorized version by decomposing the convolution into two separate layers. We refer to the first layer as depth-wise convolution, which functions as simple filtering and applies one convolution filter per input channel. According to [23], the second type is 1$\times$1 convolution, also known as point-wise convolution. It creates new features by figuring out linear combinations of the input channels, as seen in Fig. 4. The first transformer repeats this block three times, while the second transformer repeats it only once. We employ inverted residual blocks to reduce computational complexity while maintaining model accuracy. Inverted residual networks achieve a balance between model efficiency and performance.

#### Decoders

**Fig. 5:**
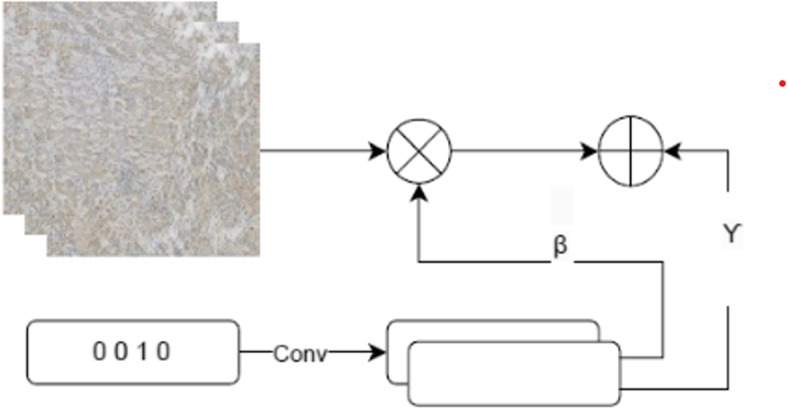
Adaptive Normalization Layer, which normalizes the features from the input image by using learnable parameters of given labels

The decoder, consisting of a convolutional transpose layer with a 3$\times$3 kernel and stride 2, follows the transformer. The first decoder consists of three upsampling layers and the second one has one upsampling layer. In this phase, we replace the normalization layers with Adaptive Instance Normalization (AdaIN) [24] instead of Instance Normalization (IN). Figure 5 shows the process of AdaIN. It adapts the output style to match the target style by normalizing the feature activations and then scaling and shifting them using learned parameters [24]. This allows for effective style transfer and enhances the visual quality of the generated images. The first generator’s transformer, which consists of three inverted residual blocks, then receives the generated features from the second generator. The decoder in the first generator similarly includes three upsampling layers, each followed by AdaIN.

Multi-scale processing, shared layers, feature integration from MobileNetV3, and advanced techniques like inverted residual blocks and AdaIN work together to make a strong framework for making realistic, high-quality images. This approach ensures that the generated images are detailed, structurally consistent, and visually appealing while optimizing model efficiency and resource usage.

### Discriminator phase

We use The discriminator of Pix2PixHD proposed in [19] because it plays a crucial role in distinguishing between real and generated images, ensuring the generator produces high-quality and realistic outputs. The model employs a multi-scale discriminator architecture, which enhances its ability to capture details at various levels of resolution. This design uses three discriminators, each operating at a different scale (original, 1/2, and 1/4 of the original resolution), allowing it to assess image quality at different spatial scales and capture both fine details and global structures, leading to more realistic image synthesis. The architecture of all discriminators utilizes the PatchGAN structure, dividing the image into overlapping patches and classifying each as real or fake, thereby focusing on the local structure of images and effectively capturing texture details. The discriminators consist of several convolutional layers with 4$\times$4 kernels and strides of 2, with the number of filters increasing progressively, starting from 64 and doubling at each subsequent layer. After each convolutional layer, we apply an instance normalization layer to provide stability during training and improve the generated images’ quality. Then we apply Leaky ReLU activation functions to introduce non-linearity and facilitate gradient flow during back-propagation.

## Loss function

The loss functions used in the proposed model are critical for training the generator and discriminator to produce high-quality, realistic images. The model utilizes a combination of adversarial loss, feature-matching loss, and perceptual loss to achieve this goal. Here, each used loss function, including its mathematical formulations and contributions to the overall training process, is illustrated.

### Adversarial loss

The training of Generative Adversarial Networks (GANs), including Pix2PixHD, relies heavily on adversarial loss [19]. This loss function ensures the generated images look identical to real ones. The adversarial loss for both the generator *G* and the discriminator *D* can be described as Eq. (1):

1 $$\begin{aligned} L_{GAN} = \mathbb {E}_{x,y}[\log (D(x,y))]+\mathbb {E}_{x}[\log (1- D(x,G(x)))] \end{aligned}$$

Here *x* represents the input image, *y* denotes the actual output image, and *G*(*x*) is the generated image. The generator *G* aims to reduce this loss whereas the discriminator *D* attempts to amplify it.

### Feature matching loss

To make the training process of GANs more stable and also to generate more quality images, Pix2PixHD uses a feature matching loss [19]. This loss ensures that the discriminator’s intermediate features extracted from real and generated images should be similar. The first and second generators calculate this loss for the generated output image, defining it as in eq.(2):

2 $$\begin{aligned} L_{FM}(G,D)&= \mathbb {E}_{x,y}\sum _{i=1}^{L}\frac{1}{N_i}[\left\| (D_i(x,y))]- D_i(x,G(x))\right\|_1 ] \nonumber \\&\quad + \mathbb {E}_{x_{s},y_{s}}\sum _{i=1}^{L}\frac{1}{N_i}[\left\| (D_i(x_{s},y_{s}))]- D_i(x_{s},G(x_{s}))\right\|_1 ] \end{aligned}$$

Where $D_{i}$ represents extracted features from the i-th layer of the discriminator, *L* indicates the overall number of layers, and $N_{i}$ represents the element count in the i-th layer. additionally $x_{s}$, and $y_{s}$ are fake low-resolution image from the second generator. The feature matching loss encourages the generator to produce images that do not look real and have similar internal representations to real images.

### Perceptual loss

We use perceptual loss to measure the difference between the real and generated images in a feature space rather than pixel space. It leverages a pre-trained network (usually VGG) to extract high-level features [25]. The perceptual loss is given by eq.(3):

3 $$\begin{aligned} L_{VGG}(G) = \mathbb \sum _{i=1}^{L}\frac{1}{N_i}[\left\| (\phi _i(y))]- \phi _i(G(x))\right\|_1 ] \end{aligned}$$

where $\phi$ represents the features extracted from the *i-th* layer of the VGG network, and *N* is the total number of layers used. This loss preserves the perceptual quality of the synthesized images by focusing on high-level features crucial for human visual perception.

### L1 loss

L1 loss, also known as mean absolute error (MAE), quantifies the pixel-by-pixel difference between the generated and real images as described in eq.(4). It is effective for preserving low-frequency information [26] and ensuring that the generated images are structurally similar to the target images.

4 $$\begin{aligned} L_{1} = \mathbb {E}_{x,y}[\left\| y - G(x)\right\|_1 ] \end{aligned}$$

where *y* is the real target image and *G*(*x*) is the generated image from the given input.

### Combined loss function

The total loss function used in Pix2PixHD is a weighted sum of the adversarial loss, feature matching loss, and perceptual loss. It can be expressed as in eq.(5):

5 $$\begin{aligned} L_{total} = \lambda _{GAN}L_{GAN}+\lambda _{VGG}L_{V\;GG}+\lambda _{FM}L_{FM}+\lambda _{L1}L_{L1} \end{aligned}$$

where $\lambda _{GAN}$, $\lambda _{FM}$, $\lambda _{VGG}$ are the weights that balance the contribution of each loss term. These weights are typically set based on empirical experimentation. A combination loss is necessary for the IHC-GAN model to balance the various demands of high-quality image generation. Each loss function addresses a specific aspect of image synthesis, and combining them ensures that the generated images meet the expectations of realism, detail preservation, and structural accuracy.

## Dataset

**Fig. 6:**
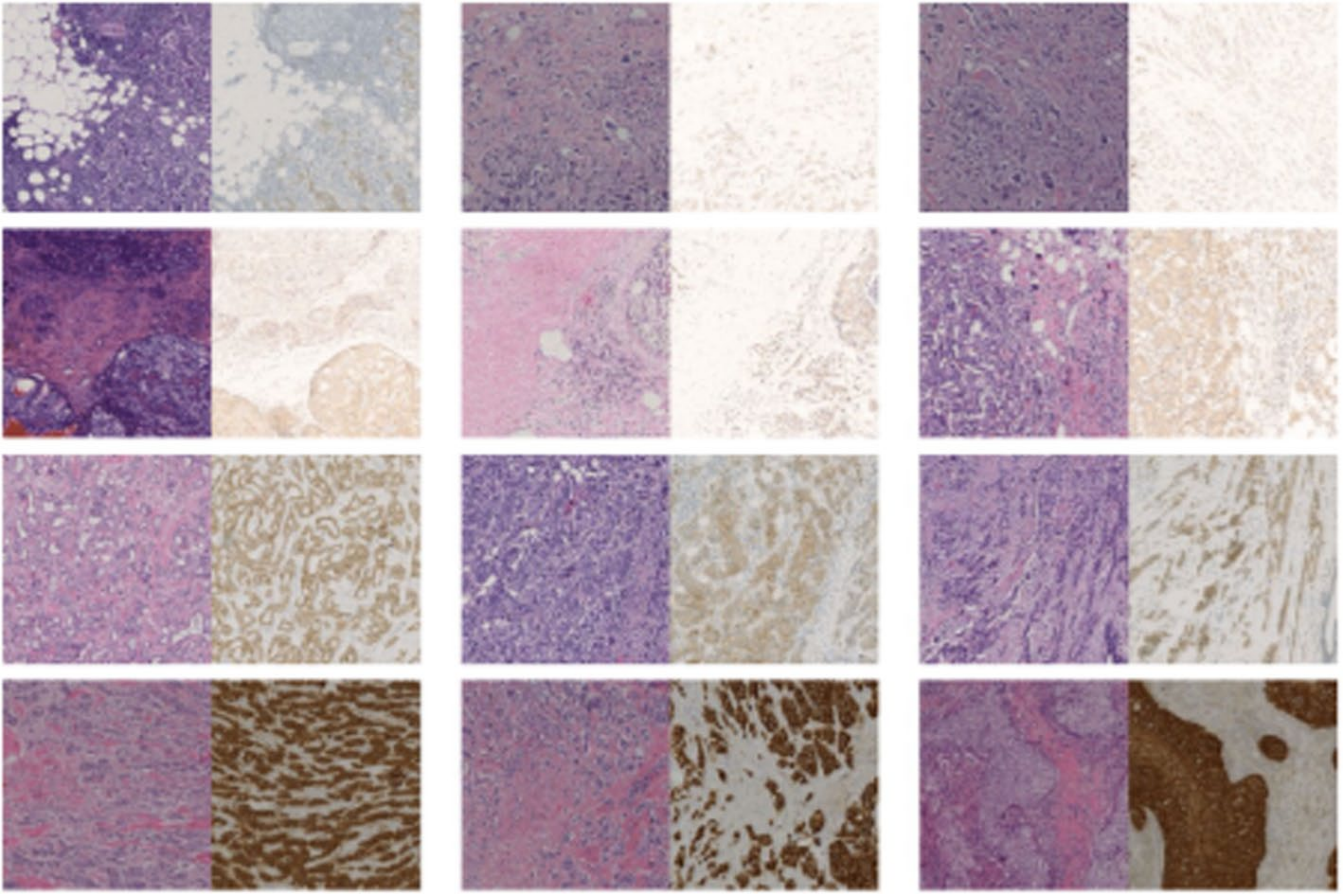
Samples of BCI dataset

We extensively evaluate the proposed IHC-GAN model using the Breast Cancer Immunohistochemical Image Generation (BCI) [13]. The dataset comprises 4870 registered image pairs, including different levels of HER2 expression (0, 1+, 2+, 3+). The data is randomly [15] distributed in a balanced manner, with approximately 70%,20%, and 10% for training, validation, and testing, respectively, resulting in 3,396 pairs for training, 977 pairs for validation, and 500 pairs for testing. A sample of the BCI dataset is presented in Fig. 6.

## Performance evaluation

Quantitative assessment of the quality of generated images poses a challenging and ongoing task. The Structural Similarity Index (SSIM) is one of the methods that can be used to compare the similarity of two images. It is employed to quantify the distortion that results in the reconstructed image against the reference image. SSIM considers structural information, brightness, and contrast; thus, it is a more perceptive model. The Structural Similarity Index Method (SSIM) can be represented through three key components, as shown in eq.(6).

6 $$\begin{aligned} SSIM=[l(x,y)]^{\alpha } \cdot [c(x,y)]^{\beta }\cdot [s(x,y)]^{\gamma } \end{aligned}$$

where *l*(*x*,*y*) describes luminance that shows the difference in brightness between corresponding points of two images. *c*(*x*,*y*) represents contrast, this is the ability of an image to differentiate the value between the darkest and the brightness of the images. *s*(*x*,*y*) describes a structure, that measures the similarity of the luminance patterns depending on two pictures. $\alpha , \beta ,$ and $\gamma$ are positive constants. Luminance, contrast, and structure can be represented mathematically using Eq. (7):

7 $$\begin{aligned} l(x,y)&=\frac{2\mu _{x}\mu _{y}+C_{1}}{\mu _{x}^{2}+\mu _{y}^{2}+C_{1}}\nonumber \\ c(x,y)&=\frac{2\sigma _{x}\sigma _{y}+C_{2}}{\sigma _{x}^{2}+\sigma _{y}^{2}+C_{2}}\nonumber \\ s(x,y)&= \frac{\sigma _{xy}+C_{3}}{\sigma _{x}\sigma _{y}+C_{3}} \end{aligned}$$

Here $\mu _{x}$ and $\mu _{y}$ are the local means of the images x and y, respectively. $\sigma _{x}$ and $\sigma _{y}$ are the standard deviations of x and y, respectively.$\sigma _{xy}$ is the cross-covariance of *x* and *y*.

PSNR is a quantitative image quality metric that is represented by the difference in pixel intensities of two pictures. SNR quantifies the quality of a signal relative to the noise by expressing the signal’s power relative to the level of noise that distorts it. PSNR is calculated from the mean squared error (MSE) between the original and the degraded images measured in dB as shown in the eq.(8):

8 $$\begin{aligned} PSNR = 10\log _{10}\left( \frac{max val^{2}}{MSE}\right) \end{aligned}$$

where maxval is the maximum pixel intensity level in the 8-bit image. MSE is the mean of the squared error between the original image and the distorted image. Metrics like the structural similarity index (SSIM) [27] and peak signal-to-noise ratio (PSNR) have been commonly used for evaluating exemplar-based [28] methods. However, we and other researchers in [29] observe that these metrics are not well-suited for assessing deep-learning models. A notable observation is that the SSIM score tends to favor blurry and smooth synthetic images, in contrast to human visual perception, which has a preference for sharper images.

Another metric used is cosine similarity, which determines how similar two vectors of features are in a multi-dimensional space by calculating the cosine of the angle between them. Consider that r and g are the two feature vectors from the real image and synthetic image. It can be represented as in eq.(9):

9 $$\begin{aligned} similarity(r,g) = cos(\theta ) = \frac{r\cdot g}{\left|| r |\right| \left|| g|\right| } \end{aligned}$$

Euclidean distance is the most basic of the measures for determining similarity distances and measures the distance between any two points in an Euclidean plane. It is given by the following Eq. (10):

10 $$\begin{aligned} d(r,g) = \sqrt{\sum _{i=1}^{n}(r_i-g_i)^2} \end{aligned}$$

The development of a perceptual metric that accurately reflects the ability of humans to quantify the similarity between two images presents a substantial challenge. This challenge has been comprehensively discussed by Zhang et. al [30], where the authors conducted a large-scale experiment using human subjective labels and addressed the key issues concerning image quality assessment. One important finding is that deep network activations are a robust perceptual similarity metric. Based on this discovery, they proposed a learned perceptual image patch similarity (LPIPS) that was achieved by affixing a linear layer to classification networks such as SqueezeNet [31], AlexNet [32], and VGG [33]. LPIPS takes two images (image patches) as input, calculates the L2 distance between the normalized deep feature representations of the input image patches, and utilizes a linear layer for estimating the perceptual judgment score.

In our evaluation of perceptual similarity between synthetic and real images, we utilize one variant LPIPS (VGG) provided by the authors [34]. A lower score in this metric indicates higher quality for synthetic images.

The Fréchet inception distance (FID) [35] is one of the sample-based evaluation metrics useful in GANs. FID is specifically meant to measure the Frechet distance between two Gaussian distributions, whereby one is the synthetic and the other the real image, as described as follows in eq.(11):

11 $$\begin{aligned} FID = \left\| \mu _y-\mu _{G(x)} \right\| ^2+Tr\left(\sum _y +\sum _{G(x)}-2\left(\sum _y\sum _{G(x)}\right)^\frac{1}{2}\right) \end{aligned}$$

where $\mu _y$ is the mean of the extracted features for the real images and $\mu _{G(x)}$ for the generated images. And $\sum _y$ and $\sum _{G(x)}$ represent the covariance of the feature maps extracted from real images as well as the generated images, respectively.

In our computation; we are comparing the FID score between fake generated and real images where the lower the FID score the better the quality of the synthetic images.

## Results and discussion

Our experiment was performed on NVIDIA Geforce Graphics 8GB GPU. Table 1 illustrates the ablation results of each stage that contributed to the development of our proposed IHC-GAN model. We make minor adjustments to the original pix2pixHD model to ensure compatibility with smaller inputs. Due to the decreased image resolution, it poses a challenge in modifying the model’s parameters without influencing the results. We limit the number of generators to a maximum of two and reduce the ResNet blocks from nine to three layers. This resulted in a reduction in size for the pix2pixHD model, which was subsequently renamed *R_Pix_HD*. However, this reduction in image resolution and increase in the number of layers led to the disappearance of significant features. Consequently, the modified model, Modified Pix2PixHD, effectively reflects our contributions but demonstrates limitations in preserving image quality. We perform and compare various configurations, including the Modified Pix2PixHD with AdaIN, Modified Pix2PixHD with AdaIN and Inverted Residual blocks (*IR*), and Modified Pix2PixHD + Classifiers with feature integration from the MobileNetV3.

**Table 1:** Ablation experiments results of each phase of the proposed model on BCI dataset

Model:	FID$\varvec{\downarrow }$	LPIPS$\varvec{\downarrow }$	PSNR$\varvec{\uparrow }$	SSIM$\varvec{\uparrow }$	MSE	CS	ED	DL-Features
Modified Pix2PixHD	0.3416	0.3751	20.12	0.3725	1368.77	0.9911	13618.72	0.8223
Modified Pix2PixHD+AdaIN	0.2311	0.3625	21.01	0.3722	1145.21	0.9906	12270.02	0.8447
Modified Pix2PixHD +AdaIN+IR	0.1776	0.3615	21.23	0.3734	1147.86	0.9904	12473.17	0.8389
Modified Pix2PixHD+ Features	0.1577	0.3604	22.25	0.3743	799.87	0.9903	10398.92	0.8464
Proposed IHC-GAN	**0.0927**	**0.3534**	**22.87**	**0.3735**	**631.52**	**0.9905**	**9443.11**	**0.8627**

**Fig. 7:**
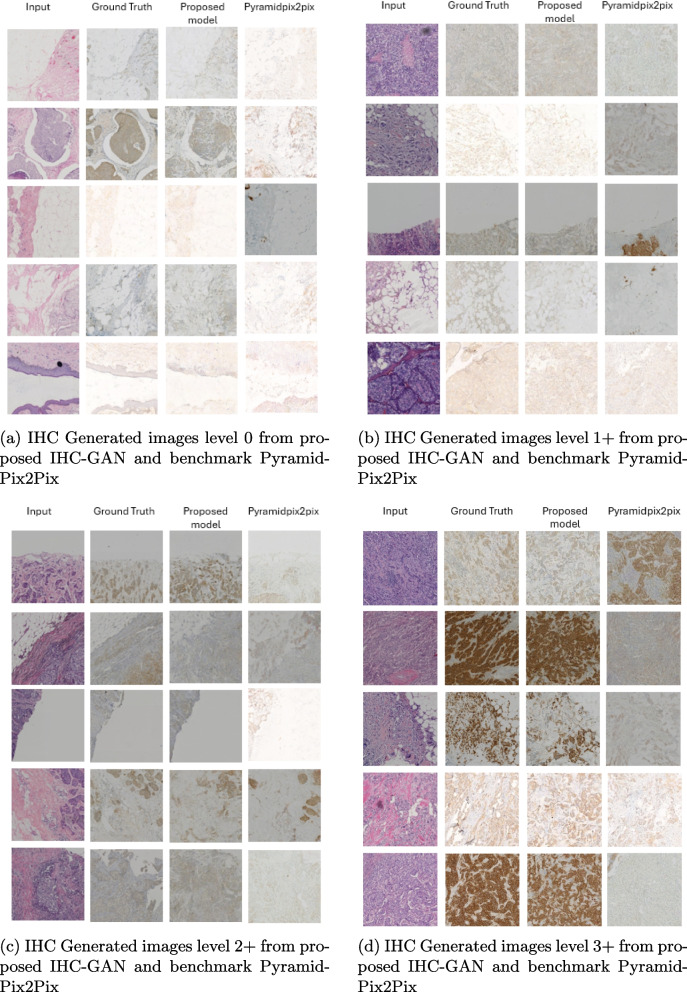
Examples of HE and IHC stain images

We use evaluation metrics such as FID, LPIPS, PSNR, SSIM, MSE, CS, ED, and DL-Features, with lower values being better for FID, LPIPS, MSE, and ED, and higher values being better for PSNR, SSIM, Cosine Similarity, and Deep Learning-Features. The proposed IHC-GAN model demonstrates superior performance across almost all evaluation metrics compared to the other configurations. Specifically, IHC-GAN achieves the lowest FID (0.0927), indicating the highest fidelity and image quality. Additionally, it attains the lowest LPIPS (0.3534), showing improved perceptual similarity to the ground truth images. In terms of reconstruction accuracy, IHC-GAN outperforms others with the lowest MSE (631.52) and ED (9443.11). Moreover, IHC-GAN achieves the highest PSNR (22.87), reflecting its superior ability to preserve image details, and high SSIM (0.3735), demonstrating excellent structural similarity to the reference images. The model also perform well in maintaining image contrast and quality as indicated by high CS (0.9905) and DL-Features (0.8627). The results of benchmark models are presented in Table 2 after down-sampling the images. As shown in Table 2, the benchmark Pix2PixHD is the most promising in all measurements (FID, LPIPS, PSNR, and SSIM). We optimize it using specifically analyzed methods for enhancement. Our proposed model outperforms the other models in all metrics except for SSIM and CS. SSIM alone is insufficient for evaluating image similarity, as indicated in [29]. The FID value is significantly higher in the PyramidPix2Pix model compared to our model, as evidenced by the images. Their images are noticeably far from the target images, as shown in Fig. 7 which displays the images generated at various levels by both the proposed model and the benchmark PyramidPix2Pix.

**Table 2:** Comparison of Evaluation Metrics (PSNR, SSIM, FID, LPIPS, MSE, Cosine Similarity, Euclidean Distance and Deep learning based Features) for Applied Techniques on the BCI Dataset

Model:	FID$\varvec{\downarrow }$	LPIPS$\varvec{\downarrow }$	PSNR$\varvec{\uparrow }$	SSIM$\varvec{\uparrow }$	MSE	CS	ED	DL-Features
AdaIN	0.5562	0.3572	20.74	0.3842	1288	0.9913	12970.07	0.8273
SPADE [36]	0.5838	0.3716	21.55	0.3784	899.83	0.9915	11214.61	0.8325
DenseNet [37]	0.4574	0.3724	20.33	0.3743	1319.90	0.9912	13297.42	0.8234
InvertedResidual Blk	0.3843	0.37	20.45	0.3845	1265.68	0.9914	13127.06	0.8244
Inverted+Dense	0.5085	0.3694	20.61	0.3834	1416.73	0.9916	13248.96	0.8266
SPADE+Dense	0.4184	0.3724	21.53	0.3762	915.43	0.9911	11343.03	0.8284
SPADE+inverted	0.6450	0.3635	22.16	0.3933	792.24	0.9916	10478.93	0.8426
SPADE+inverted+Dense	0.6588	0.3756	22.02	0.3926	840.33	0.9914	10744.03	0.8323
Pix2Pix	0.4889	0.4029	19.56	0.3552	1478.94	0.9906	14312.78	0.7973
PyramidPix2Pix	1.2652	0.3979	20.65	0.3725	1265.57	0.9915	12881.87	0.8012
Pix2PixHD	0.7898	0.3678	20.86	**0.3944**	1154.55	**0.9917**	12498.61	0.8261
Proposed IHC-GAN	**0.0927**	**0.3534**	**22.87**	0.3735	**631.52**	0.9905	**9443.11**	**0.8627**

Dual-scale generators and multi-scale discriminators are employed to stabilize the training process and the model performance, effectively reducing the risk of mode collapse. Additionally, different loss functions, such as feature matching loss and perceptual loss, are utilized to further enhance the training stability and improve the quality of the generated images. The balanced distribution manner of the dataset allows the model to learn more generalized features, which reduces the risk of overfitting. Furthermore, the augmentations help the model become more robust by exposing it to a wider range of data patterns, thereby enhancing its ability to perform well on unseen data. The risk of overfitting has been reduced and the model’s generalisation capabilities have been preserved by implementing these strategies. The use of Adaptive Instance Normalization (AdaIN), which employs style transfer techniques, directs the generator to produce specific stain patterns, a task that cannot be achieved without utilizing a classifier. This approach results in generated images that closely resemble the target outputs, which is evidenced by improved Fréchet Inception Distance (FID) scores. Additionally, the integration of fine-grained features into the generator, along with shared layers between two generators, contributes to better generalization of features, thereby enhancing the consistency and quality of the generated images. These enhancements are reflected in the observed increases in Mean Squared Error (MSE) and Peak Signal-to-Noise Ratio (PSNR) values.

Due to limited computational resources, we were obligated to downscale the input images to a lower resolution. This led to the loss of some fine details and features in the generated images. High-resolution images typically contain more complex patterns and structural information, and downscaling compromises the ability of the model to fully capture and translate all of these details accurately. As a result, some critical image features were lost during the generation and translation process, potentially affecting the overall quality of the generated images.

The MobileNetV3 classifier used in our model to determine HER2 levels has a small percentage error. Even though the classifier achieves high accuracy, any misclassification can lead to errors in the staining process of the translated image. This is because the classifier provides the guidance needed to apply the correct stain level to the generated IHC image. Any misstep in classification affects the accuracy of the staining and, subsequently, the overall quality and reliability of the translated images.

One of the limitations of the current IHC-GAN model is its sensitivity to the quality of the input data. Poorly labelled or noisy data can lead to suboptimal training and unreliable outputs. Despite employing various techniques to mitigate these issues, this implementation did not incorporate the concept of stochastic resonance. Stochastic resonance, which involves the introduction of controlled noise to enhance weak signals, has been shown to improve model robustness and performance in similar contexts, such as medical image segmentation and object-tracking applications [38–41].

**Fig. 8:**
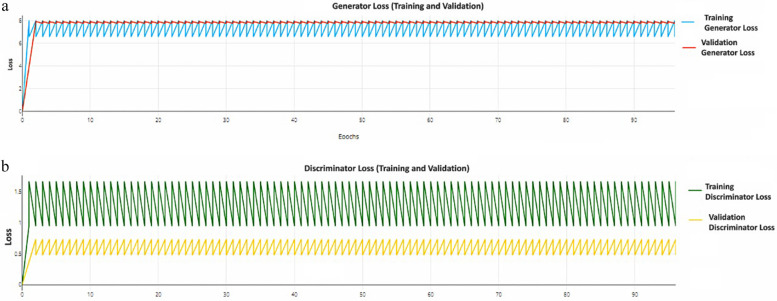
Training and Validation Loss for the Discriminator and Generator

Figure 8 illustrates the loss trends for both the discriminator and generator during the training and validation phases over a series of epochs. In the discriminator loss graph, the training and validation losses, represented by the green and yellow curves respectively, show oscillatory patterns that stabilize around consistent values. This behavior is typical in generative adversarial Networks (GANs) due to the adversarial dynamics between the discriminator and the generator. The oscillations suggest that the discriminator is continuously adjusting its parameters as it attempts to distinguish between real and generated data, while the stability of the loss values indicates that it is learning effectively without suffering from instability or collapse. The stability observed after the initial spikes demonstrates that both the discriminator and generator have reached equilibrium, which is crucial for effective GAN training. This overall pattern of loss convergence is a positive indicator that the model is learning properly.

Table 3 compares the complexity, measured in the number of parameters, between different models: PyramidPix2Pix, Pix2PixHD, and the proposed IHC-GAN. PyramidPix2Pix is the least complex, with 11.38 million parameters. In contrast, Pix2PixHD is significantly more complex, with 186.92 million parameters. The proposed model strikes a balance between these two, with 26.77 million parameters, indicating a moderate level of complexity. This suggests that the proposed IHC-GAN aims to optimize performance while maintaining a manageable number of parameters, potentially enhancing efficiency and computational feasibility. Additionally, the model achieves faster convergence than the others. Moreover, the model requires approximately 41.13 GFLOPs for a single forward pass. Considering both the forward and backward passes during training, the total FLOPs are estimated as 82.26 GFLOPs. This value indicates that our model maintains a reasonable computational complexity relative to its size and performance. The reported GPU memory usage is 1.28 GB of VRAM. During training, the model utilizes approximately 11.11 GB of system RAM, which is within acceptable limits for deep-learning models of this size. Each epoch takes an average of 9.25 minutes. For 200 epochs, the total training time is 30.83 hours. This training time demonstrates that our model converges efficiently, despite the relatively large parameter count.

**Table 3:** Comparison between complexity analysis between benchmarks and proposed model

Model	No. of parameters
PyramidPix2Pix	11.38 M
Pix2PixHD	186.92 M
Proposed IHC-GAN	26.77 M

The IHC-GAN model has fewer training parameters compared to other GAN models, making it lighter and enabling it to complete training in approximately 9 minutes per epoch. Utilizing parallel computation can further boost the model’s speed and efficiency while maintaining the same number of training parameters. By distributing the workload across multiple processing units, parallel computation can significantly reduce the total training time and optimize resource utilization. Previous research has demonstrated the effectiveness of parallelization techniques in accelerating computationally intensive tasks [42–45].

**Fig. 9:**
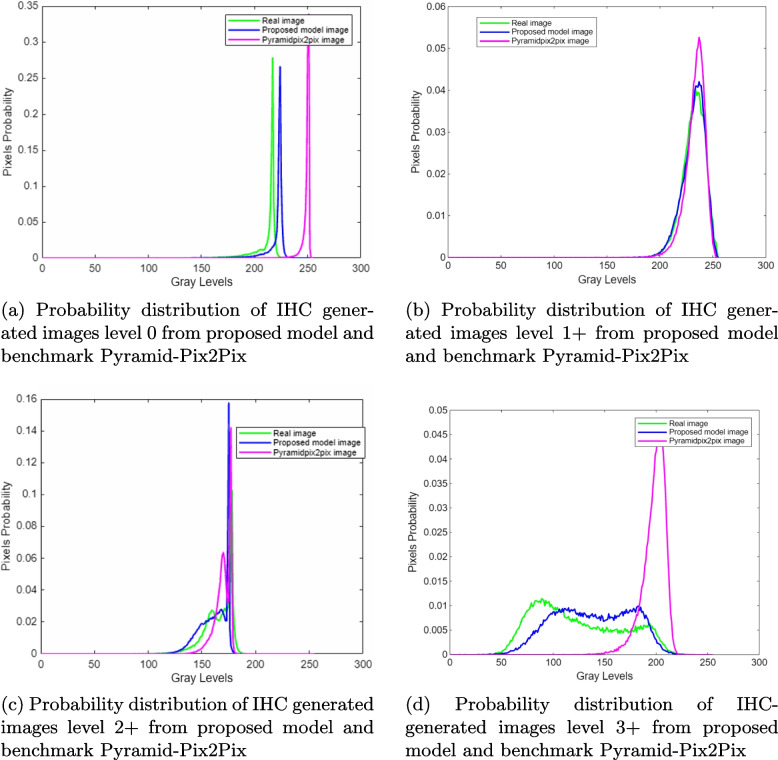
Examples of HE and IHC stain images

Moreover, the probability distribution of generated images of our model is illustrated below in Figs. 9, which shows that the probability distribution of our model is very close to the actual images, more than the benchmark pyramidPix2Pix.-

## Conclusion

IHC staining is a labor-intensive process that requires a high level of expertise and is necessary for the diagnosis and treatment planning of breast cancer. We develop a new IHC-GAN approach for generating high-quality IHC-stained images from down-sampled H&E-stained images to address these issues. The proposed method has low complexity and employs two different-scale generators: inverted residual networks (AdaIN) and feature combinations. The results demonstrate encouraging image quality and high structural coherence. Additionally, the model variations achieve lower FID values when compared to SOTA methods Pix2Pix and PyramidPix2Pix, indicating higher similarity to the target images with values of 0.0927 FID, 22.87 PSNR, and 0.3735 SSIM. Furthermore, the presented model requires a significantly smaller number of trained parameters than the Pix2PixHD. This model’s significance lies in its ability to reduce the costs and manpower needed for traditional HER2 assessment methods, facilitating timely and accurate treatment decisions in breast cancer care. By providing an efficient and reliable alternative, our IHC-GAN model can significantly enhance the diagnostic process, making it more accessible and less demanding for healthcare providers.

## Data Availability

Liu et al. provided the dataset, “BCI: Breast Cancer Immunohistochemical Image Generation Through Pyramid Pix2pix”, which is accessible online at https://bupt-aicz.github.io/BCI/.
